# Uremic Clearance Granules Regulate Immune Equilibrium via Gut Microbiome to Alleviate Chronic Renal Failure

**DOI:** 10.34133/bmr.0342

**Published:** 2026-03-30

**Authors:** Qian Huang, Zhuowen Liang, Yuqing Cui, Jianxin Diao, Tianshu Zhou, Lei Shi, Zhixin Deng, Rushang Wang, Haitao Yuan, Kun Chen, Ying Du, Ali Chen, Jiayun Chen, Wei Xiao

**Affiliations:** ^1^ Key Laboratory of Glucolipid Metabolic Disorder, Ministry of Education, Guangdong Pharmaceutical University, Guangzhou, Guangdong 510006, China.; ^2^ School of Traditional Chinese Medicine, Southern Medical University, Guangzhou, Guangdong 510515, China.; ^3^ Department of Faculty of Education, City University of Hong Kong, Hong Kong, China.; ^4^ Consun Pharmaceutical Group Limited, Guangzhou, Guangdong 510006, China.; ^5^ Center for Drug Research and Development, Guangdong Provincial Key Laboratory of Pharmaceutical Preparations Research and Evaluation, Guangdong Pharmaceutical University, Guangzhou 510006, China.

## Abstract

Chronic renal failure (CRF) is the common end point of various chronic kidney diseases, and there is currently no specific drug for CRF. Effectively halting its progression remains a clinical challenge. Gut microbiota disorders are a key factor influencing immune dysfunction in chronic kidney disease patients. Intervening in gut microbiota to improve immune regulatory function in patients could serve as a new strategy for treating CRF with Traditional Chinese Medicine. Uremic Clearance Granules (UCG), a Traditional Chinese Medicine formulation, effectively attenuate CRF progression, but their active components and mechanisms remain undefined. This study investigates how UCG mitigate CRF via coordinated regulation of gut microbiota, metabolites, and the T helper 17 cells / regulatory T cell axis. Using an adenine-induced CRF mouse model, we combined gut microbiota depletion, fecal microbiota transplantation, 16*S* rRNA sequencing, and metabolomics to delineate the gut–kidney interactions underlying UCG efficacy. Flow cytometry quantified immune cell profiles in blood, and microbial intervention experiments verified the therapeutic role of *Bifidobacterium animalis* (*B. animalis*). In this study, we found that UCG treatment alleviated renal injury, reduced intestinal permeability, and up-regulated intestinal barrier markers. Microbiota depletion and fecal microbiota transplantation demonstrated that UCG’s renoprotective effects depend on gut microbial modulation. Specifically, UCG ameliorates CRF through gut–kidney axis remodeling by enhancing *B. animalis* abundance and sophocarpine, thereby rebalancing T helper 17/regulatory T immunity and preserving renal function. These findings identify a microbiota-dependent immunometabolic mechanism for UCG and highlight a potential therapeutic strategy for CRF via the drug–microbiota axis.

## Introduction

Chronic kidney disease (CKD) is a rapidly escalating global health burden, projected to become the fifth leading cause of mortality worldwide by 2040 [1]. It represents a continuum of chronic renal injury and dysfunction arising from diverse etiologies and is marked by progressive declines in glomerular filtration rate, tubular cell apoptosis, and loss of renal function, ultimately culminating in chronic renal failure (CRF) [2]. Although multiple targeted therapies are under clinical investigation to slow CKD progression [3], their long-term administration often leads to serious adverse effects, including electrolyte imbalance, metabolic disturbance, allergic reactions, infections, and hematologic toxicity [4]. These limitations underscore the urgent need for safer, multitarget therapeutic approaches capable of achieving sustained renal protection. In this context, Traditional Chinese Medicine (TCM) offers a holistic and individualized paradigm that emphasizes syndrome differentiation and integrative regulation [5,6].

Owing to its favorable safety profile, clinical efficacy, and multimodal benefits, elucidating the molecular mechanisms and regulatory targets of TCM-derived herbal formulations holds marked promise for developing safer and more effective interventions for CKD. Given accumulating evidence that immune dysregulation drives CKD progression, the immunomodulatory potential of TCM formulations warrants particular attention. Renal function is tightly intertwined with immune homeostasis. Dysregulation of immune balance is a central driver of CRF progression, exacerbating metabolic disturbances, oxidative stress, and uremic toxin accumulation in renal tissues [7]. The development of CKD is marked by extensive inflammatory cell infiltration, including aberrant T cell activation and macrophage polarization, which together disrupt the renal immune microenvironment [8]. Among these immune components, T lymphocytes are pivotal regulators of renal immunity, orchestrating the activity of CD4^+^ and CD8^+^ subsets to maintain immunological equilibrium [9]. Consequently, T cell-mediated immune imbalance is closely linked to CRF pathogenesis. Restoring renal immune homeostasis through targeted modulation of T-lymphocyte subsets involved in inflammation may therefore represent an effective strategy to mitigate CRF progression. In particular, the dynamic equilibrium between proinflammatory T helper 17 (Th17) cells and immunosuppressive regulatory T (Treg) cells has emerged as a critical determinant of renal inflammatory homeostasis.

At the same time, mechanistic studies further reveal that gut microbiota-mediated modulation of renal immune homeostasis represents a key pathway through which TCM exerts renoprotective effects [10,11]. Emerging evidence underscores a strong bidirectional relationship between the gut microbiota and CKD [12]. Nutritional and pharmacological interventions targeting the gut microbiota have demonstrated considerable therapeutic potential in alleviating CKD and its metabolic complications [13]. Distinct microbial signatures may serve as biomarkers for disease progression, offering novel avenues for intervention [14]. Notably, *Bifidobacterium animalis* (*B. animalis*) abundance is markedly reduced in patients with kidney disease [15,16]. Conversely, microbial dysbiosis contributes to immune dysfunction and chronic systemic inflammation in CKD [17], suggesting that microbiota-targeted restoration of immune equilibrium could serve as an effective therapeutic strategy. Thus, microbiota-driven regulation of T cell balance—particularly the Th17/Treg axis—has emerged as a central mechanism for mitigating CKD progression.

**Fig. 1.**
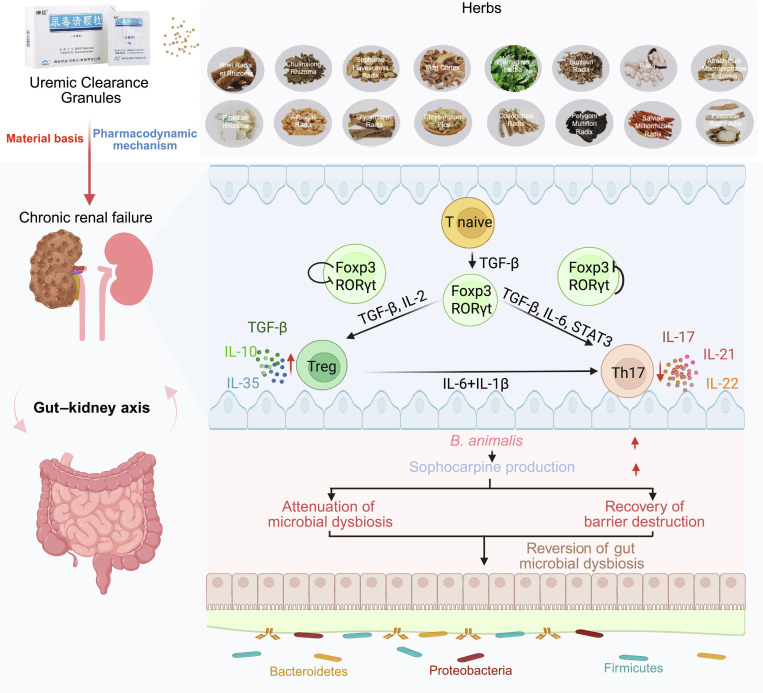
UCG ameliorates CRF through gut–kidney axis remodeling by enhancing *B. animalis* abundance, thereby rebalancing Th17/Treg immunity and preserving renal function.

Current studies have shown that TCM intervention in gut microbiota can achieve immunomodulation and thereby enhance collective immune function and maintain the body’s healthy state. Uremic Clearance Granules (UCG) are a clinically approved TCM preparation with immunomodulatory and anti-inflammatory properties that has been shown to relieve uremia symptoms, delay disease progression, and improve quality of life in patients with CKD. It has shown marked clinical efficacy in the treatment of CRF [18,19]. However, the pharmacological mechanisms underlying their efficacy remain poorly defined. Given its clinical applicability and therapeutic potential, UCG represents an ideal candidate for investigating the interplay between gut microbiota, immune regulation, and CKD progression. This study aims to elucidate the mechanisms by which UCG modulates the gut microbiota and restores the Th17/Treg immune balance, thereby providing new insights into the development of microbiota-targeted therapies for CKD (Fig. 1).

## Materials and Methods

### Chemicals and reagents

UCG was supplied by Consun Pharmaceutical Co., Ltd. Losartan was purchased from Hangzhou MSD Pharmaceutical Co., Ltd. Adenine diet was purchased from Xiaoliyoutai Pharmaceutical Co., Ltd. *B. animalis* (BNCC185341) was obtained from the Beina Biotechnology Company.

### Preparation and quality control of UCG

The chemical constituents of UCG are listed (Table S1). Qualitative and quantitative analysis of paeoniflorin, the main active compound in UCG, was performed using liquid chromatography-tandem mass spectrometry. By matching the retention time of paeonol target compound with that of the reference standard, clear identification was achieved (Fig. S1).

Three grams of UCG was accurately weighed (batch numbers 2401004, 2401005, and 2401006); 25 ml of water was added, shaken well, and treated with ultrasound for 30 min. The solution was centrifuged at 4,000 rpm for 10 min, and 5 ml of the supernatant was collected and applied to a polyamide column (30 to 60 mesh, 1 cm × 20 cm). It was eluted with water; 25 ml of the eluate was precisely collected, shaken well, and filtered, and the filtrate was taken as the test solution, with paeoniflorin as the reference standard (National Institutes for Food and Drug Control, batch number 110736-202246, purity 96.7%). The chromatographic separation was performed using a C18-bonded silica gel column (250 mm × 4.6 mm, 5 μm), and acetonitrile-0.05% potassium dihydrogen phosphate solution (15:85, v/v), isocratically eluted for 30 min. Flow rate: 1 ml/min, column temperature: 30 °C, injection volume: 20 μl, and detection wavelength: 230 nm.

### Serum pharmacochemistry analysis

Normal C57BL/6J mice were acclimatized for 1 week before the experiment. The mice were administered UCG at a dose of 4.2 g/kg/d at a fixed time daily for 1 week. Blood samples were collected 2 h after the last administration, and the plasma was stored at −80 °C for component analysis by Shanghai OE Biotech. Co., Ltd

A Thermo-Orbitrap-QE HF system was used to detect the chemical constituents of UCG, blank serum, and drug-containing serum. The chromatographic separation was performed using a column (ACQUITY UPLC HSS T3, 100 mm × 2.1 mm, 1.8 μm). Mobile phase A consisted of 0.1% formic acid in water, and mobile phase B consisted of acetonitrile. Mass spectrometry signals of the samples were detected in both positive and negative ionization modes. The raw data were normalized using the metabolomics software XCMS v4.5.1, and compounds were identified using a TCM database.

### Network pharmacology study

The bioavailable compounds identified in mouse serum were considered as the active components of UCG. The target proteins of the active compounds were obtained from the TCM Systems Pharmacology Database. The GeneCards database was utilized to predict targets related to CRF. The intersection of these targets was collected, and Gene Ontology (GO) and Kyoto Encyclopedia of Genes and Genomes (KEGG) pathway analysis were performed, with a significance level of *P* < 0.05. By comparing the results of integrated pharmacological network analysis with subsequent experimental data, the mechanism of UCG in the treatment of CRF was investigated.

### Animal experiments on the treatment of CRF by UCG

C57BL/6 male mice (8 weeks old, weighing 20 to 22 g) were purchased from Guangzhou Ruige Laboratory Animal Co., Ltd. All animal experiments were approved by the Animal Care and Use Professional Committee of Guangdong Pharmaceutical University (Approval No. *gdpulacspf*2022503) and strictly followed the National Institutes of Health Guide for the Care and Use of Laboratory Animals.

The mice were housed in a specific pathogen-free (SPF) environment and randomly assigned to 6 groups (*n* = 8). The control group continued a normal diet throughout the study, and the CRF group was induced by a 0.1% adenine diet for 8 weeks. At week 4, blood urea nitrogen (BUN), serum creatinine (Scr), and urinary protein levels were measured to confirm the establishment of CRF. The CRF group was further subdivided into 5 treatment groups: CRF, Losartan, L-UCG, M-UCG, and H-UCG. The Losartan group received a daily oral dose of 7.6 mg/kg, while the UCG groups were administered low (2.1 g/kg/d), middle (4.2 g/kg/d), or high (8.4 g/kg/d) doses by gavage. At the end of the experiment, urine and fecal samples and serum, kidney, and colon tissues were collected for analysis.

### Animal experiments of gut microbiota depletion

Antibiotic treatment (ABX) was used to deplete the gut microbiota. Mice were administered a mixture of antibiotics (vancomycin, 0.5 mg/ml; neomycin sulfate, 1 mg/ml; metronidazole, 1 mg/ml; and ampicillin, 1 mg/ml) via oral gavage [20].

The mice were housed in an SPF environment and randomly assigned to 5 groups (*n* = 8). The control group continued a normal diet throughout the study, and the CRF group was induced by a 0.1% adenine diet for 8 weeks. At week 4, CRF mice were divided into 4 groups: CRF, UCG, ABX, and ABX+UCG. The ABX and ABX+UCG groups received daily ABX treatment for 4 weeks to eliminate gut microbiota, while the UCG and ABX+UCG groups were also given UCG solution (4.2 g/kg/d) by gavage. At the end of the experiment, serum and kidney tissues were collected for analysis.

### Animal experiments of fecal microbiota transplantation

The mice were housed in an SPF environment and randomly assigned to 6 groups (*n* = 8). The control group continued a normal diet throughout the study, and the CRF group was induced by a 0.1% adenine diet for 8 weeks. At week 4, CRF mice were divided into 5 groups: CRF, UCG, fecal microbiota transplantation (FMT)-Control, FMT-CRF, and FMT-UCG. The FMT-Control, FMT-CRF, and FMT-UCG groups were treated with ABX for 3 consecutive days to eliminate intestinal microbiota, followed by administration of 0.2 ml of the fecal suspension for 4 weeks. For FMT experiments in CRF mice, fecal samples from Control, CRF, and UCG-treated mice were resuspended in phosphate-buffered saline at a concentration of 100 mg/ml [21]. At the end of the experiment, serum and kidney tissues were collected for analysis.

### Bacterial strain cultivation and intervention

*B. animalis* was cultured in BBL beef broth liquid medium in a 37 °C incubator for 48 h. The suspension was centrifuged at 4 °C at 8,000 rpm for 30 s, and the precipitate was resuspended in sterile phosphate-buffered saline containing 20% glycerol to prepare a bacterial suspension with a final concentration of 1 × 10^8^ CFU/ml. C57BL/6J mice were fed a 0.1% adenine diet for 4 weeks to establish a chronic kidney failure model. For *B. animalis* intervention, we administered *B. animalis* by gavage (0.2 × 10^8^ per mouse) continuously for 4 weeks.

### Biochemical indicator analysis

Creatinine, protein, and urea nitrogen levels were measured according to the manufacturer’s instructions (Nanjing Jiancheng Bioengineering Institute, China). Optical density was determined, and the levels of these indicators were calculated.

### Histological analysis

Mouse kidney tissues were fixed with 4% paraformaldehyde, dehydrated, and paraffin embedded. Serial sections 5 μm thick were cut from each block, placed on glass slides, and then stained with hematoxylin and eosin (H&E) to observe tissue pathological changes. Masson’s trichrome and periodic acid-Schiff (PAS) staining were performed to evaluate renal fibrosis. Mouse colon tissues were stained with H&E to observe tissue pathological changes. Alcian Blue and wheat germ agglutinin staining were used to examine goblet cell numbers.

### Immunofluorescence

Kidney specimens were cryosectioned into 5-μm slices and subjected to antigen retrieval with citrate buffer (pH 6.0). Following blocking, the tissue sections were incubated overnight with primary antibodies against *Foxp3* (Affinity, AF6544, 1:500) and *RORγt* (Abcam, ab207082, 1:3,000) at 4 °C for 12 to 16 h. Secondary detection was performed using species-matched fluorescent conjugates (Alexa Fluor 594- and Alexa Fluor 488-labeled anti-rabbit immunoglobulin G, Proteintech, CTK0101, 1:400). The nuclei were visualized using 40,6-diamidino-2-phenylindole (Biosharp, BS097). The sections were examined, and photomicrographs were captured using a fluorescence microscope.

### Gut microbiota profiling by 16*S* rRNA sequencing

Fecal samples were sent to Majorbio Co., Ltd (Shanghai, China) for sequencing. Bacterial DNA was extracted for polymerase chain reaction (PCR), and details of the microbial quantification strategy are provided in the supplementary materials (Supplementary Materials).

### Nontargeted metabolomics

Untargeted metabolomic analysis of mouse feces was performed via liquid chromatography-tandem mass spectrometry. The data were imported into Progenesis QI (Waters Corporation, Milford, USA) for multivariate statistical analysis [22]. The experimental steps for metabolomics are outlined in the supplementary methods (Supplementary Materials).

### Assessment of intestinal permeability

After a 4-h fasting period, mice were orally administered 0.5 mg/g fluorescein isothiocyanate (FITC)-dextran (4 kDa; Sigma-Aldrich). Four hours later, blood serum was collected. A standard curve was prepared using serial dilutions of FITC-dextran standards (50, 25, 12.5, 6.25, 3.125, 1.56, 0.78, 0.39, 0.19, and 0 mg/ml). In a black, opaque 96-well plate, 100 μl of each sample and standard were added, with ultrapure water used as a blank control. Fluorescence was measured using a fluorescence microplate reader, with an excitation wavelength of 490 nm and an emission wavelength of 530 nm [23].

### Quantitative real-time PCR

Total RNA was extracted from tissues using TRIzol reagent (Invitrogen, Thermo Fisher Scientific, USA) following the manufacturer’s protocol [24]. The RNA concentration and quality were determined using a nanodrop. The RNA was reverse transcribed into cDNA using an APExBIO kit (K1074-100). PCR was performed with an APExBIO kit (K1070-500) under the following reaction conditions: denaturation at 95 °C for 15 s, annealing at 60 °C for 30 s, and extension at 95 °C for 1 min, with a cycle number of 40 using a QuantStudio 1 Plus Real-Time PCR Instrument. Target genes were synthesized by Wuhan Qingke Biotechnology Co., Ltd. (Table S2). Gene expression was normalized to *β-actin*, and statistical analysis was conducted using the 2^−ΔΔCt^ method.

### Flow cytometric analysis of *RORγt* and *Foxp3*

Single-cell suspensions of peripheral blood were prepared under sterile conditions. Th17 cells were labeled with CD45-APC, CD3-FITC, CD4-PE, and *IL-17*A-PE, while Treg cells were labeled with CD45-APC, CD3-FITC, CD4-PE, CD25-APC, and *Foxp3*-BV421[25]. The cell surface was stained with *IL-17*A monoclonal antibody (BioLegend, 506903) or *Foxp3* monoclonal antibody (Invitrogen, 48-5773-80). Cells were then fixed, permeabilized, and subjected to intracellular staining. Th17 cells and Treg cells were analyzed by flow cytometry.

### Statistical analysis

Statistical analysis was performed using GraphPad Prism software (version 12.0.0). All data are presented as means ± SEM and were compared using 2-tailed unpaired Student *t* test or 1-way analysis of variance with Tukey post hoc tests. Statistical significance was defined as *P < 0.05, **P < 0.01, and ***P < 0.001, with ns indicating no significant difference.

## Results

### Analysis of blood components following UCG

**Fig. 2.**
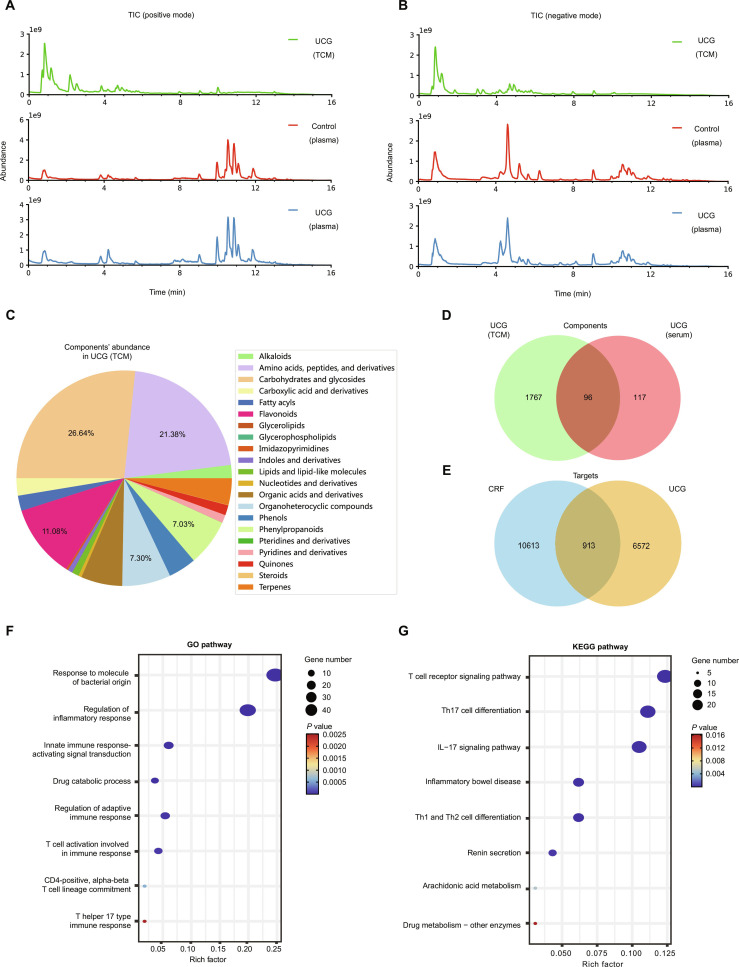
Pharmacokinetic profile of UCG granules. (A) Total ion chromatogram (TIC) of UCG and serum samples in positive ion mode. (B) TIC of UCG and serum samples in negative ion mode. (C) Classification and content distribution of UCG original components. (D) Venn diagram of original components and bioavailable compounds of UCG. (E) Venn diagram of bioavailable compounds and CRF targets. (F) GO enrichment analysis of intersection targets. (G) KEGG enrichment analysis of intersection targets.

To systematically analyze the chemical constituents of UCG extract and its metabolic profile in vivo, we performed nontargeted identification of chemical constituents in both positive and negative ion modes, identifying a total of 1,863 compounds (Fig. 2A and B). Among these, the top 5 classes based on content were sugars and glycosides (26.64%), amino acids and peptides (21.38%), flavonoids (11.08%), organic heterocyclic compounds (7.30%), and coumarins (7.03%) (Fig. 2C). Next, we compared blank serum and medicated serum, identifying 213 compounds of UCG for investigating the bioavailable compounds of UCG. Among these, 96 compounds were identified as original components of UCG entering the bloodstream, and 117 were identified as metabolites (Fig. 2D). The major bioavailable compounds included guanine, baicalein, chrysophanol, oxymatrine, sophoranone, daidzein, 8-isopentyldaidzein, genistin, baicalin-7-glucuronide, 18α-glycyrrhetinic acid, wogonoside, glycyrrhetinic acid, paeonolide, albiflorin, and 6-hydroxyapiolin-7-glucuronide-(1->2)-glucuronide. (Table S3).

Based on the bioavailability results of UCG, a total of 7,485 potential targets were predicted from the bioavailable compounds. To further screen targets related to CRF, we matched these compounds against a known dataset of 11,526 CRF-related targets, identifying 913 overlapping targets (Fig. 2E). Subsequently, we conducted GO and KEGG enrichment analyses on these overlapping targets to explore the functional mechanisms of UCG. The GO enrichment analysis demonstrated that UCG exerts its therapeutic effects on CRF primarily through biological processes such as bacterial molecular responses, inflammatory responses, drug metabolism, and immune regulation. Among these, bacterial molecular responses and immune regulatory processes showed higher confidence levels (Fig. 2F). Furthermore, the KEGG pathway enrichment analysis highlighted that inflammatory bowel disease and T cell immune responses are critical signaling pathways through which UCG alleviate CRF (Fig. 2G).

Therefore, to investigate the mechanism of action of UCG, we noted that the gut microbiota plays a crucial role in the development and regulation of the immune system. For instance, certain commensal bacteria promote the formation of the intestinal epithelial barrier, preventing pathogen invasion. Microbial metabolites, such as biogenic amines, modulate the activity of immune cells and promote anti-inflammatory responses [26]. Based on these findings, we hypothesize that UCG may alleviate CRF through a mechanism involving gut microbiota participation in the body’s immune response. Further experiments will aim to verify whether UCG exerts its therapeutic effects on CRF through a mechanism based on gut microbiota.

### UCG ameliorates CRF in adenine-induced mouse

**Fig. 3.**
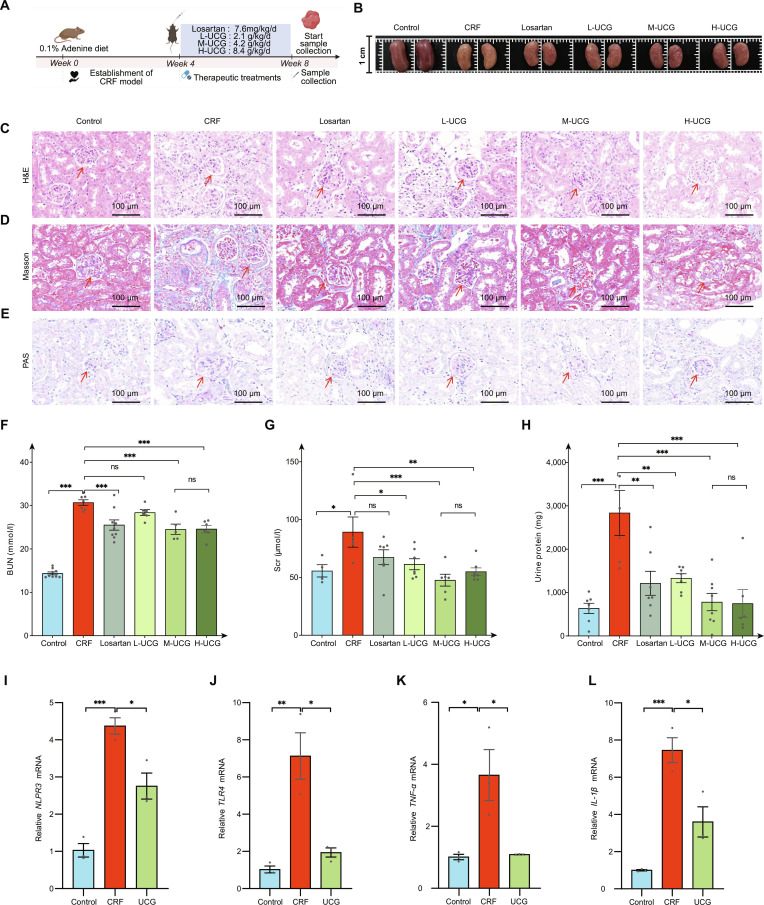
UCG attenuates renal injury in CRF mice. (A) Experimental timeline: Mice were fed an adenine diet, followed by 4 weeks of UCG treatment. (B) Renal morphology. (C) H&E staining of the kidney, and the representative areas were highlighted by arrows (scale bar = 100 μm). (D) Masson staining of the kidney, and the representative areas were highlighted by arrows (scale bar = 100 μm). (E) PAS staining of the kidney, and the representative areas were highlighted by arrows (scale bar = 100 μm). (F) Serum BUN levels. (G) Scr levels. (H) Twenty-four-hour urinary protein. (I to L) mRNA expression of *NLPR3*, *TLR4*, *TNF-α*, and *IL-1β* in kidney tissue. *P < 0.05, **P < 0.01, ***P < 0.001. ns, not significant.

To validate the efficacy of UCG in improving CRF, we designed the following experiments. First, we induced CRF in mice using a 0.1% adenine diet for 8 weeks, successfully establishing the chronic kidney failure model (Fig. 3A). Subsequently, mice were treated with different doses of UCG (low-dose L-UCG, medium-dose M-UCG, high-dose H-UCG) from 4 to 8 weeks. Through comparison with the model group, we observed that UCG treatment significantly alleviated renal abnormalities (Fig. 3B). In the pathological studies, we found that mice in the CRF group exhibited obvious renal glomerular atrophy and tubular dilation (Fig. 3C), accompanied by excessive collagen deposition (Fig. 3D), thickening of the glomerular basement membrane (Fig. 3E), and promotion of renal fibrosis development. These pathological changes indicated that the model group mice indeed suffered from marked renal damage.

Subsequently, we measured the levels of serum Scr (Fig. 3F), BUN (Fig. 3G), and urinary protein (Fig. 3H) to assess renal function. Compared to the model group, these renal function parameters significantly decreased after UCG treatment (*P* < 0.05), indicating that UCG effectively alleviated renal injury. In addition, treatment with L-UCG, M-UCG, and H-UCG all reduced the degree of renal degeneration, with the M-UCG showing the best therapeutic effect, providing a reference for future studies.

We further detected the gene expression levels of inflammatory factors *NLPR3* (Fig. 3I), *TLR4* (Fig. 3J), *TNF-α* (Fig. 3K), and *IL-1β* (Fig. 3L) to investigate the anti-inflammatory effects of UCG. The expression levels of these inflammatory factors were significantly down-regulated in the UCG-treated group (*P* < 0.05) compared with the CRF group, suggesting that UCG exerted renoprotective effects by suppressing inflammatory responses. In summary, these experimental results confirmed the effectiveness of UCG in improving chronic kidney failure and revealed its potential mechanisms of action.

### UCG restored intestinal mucosal barrier disruption in CRF mice

**Fig. 4.**
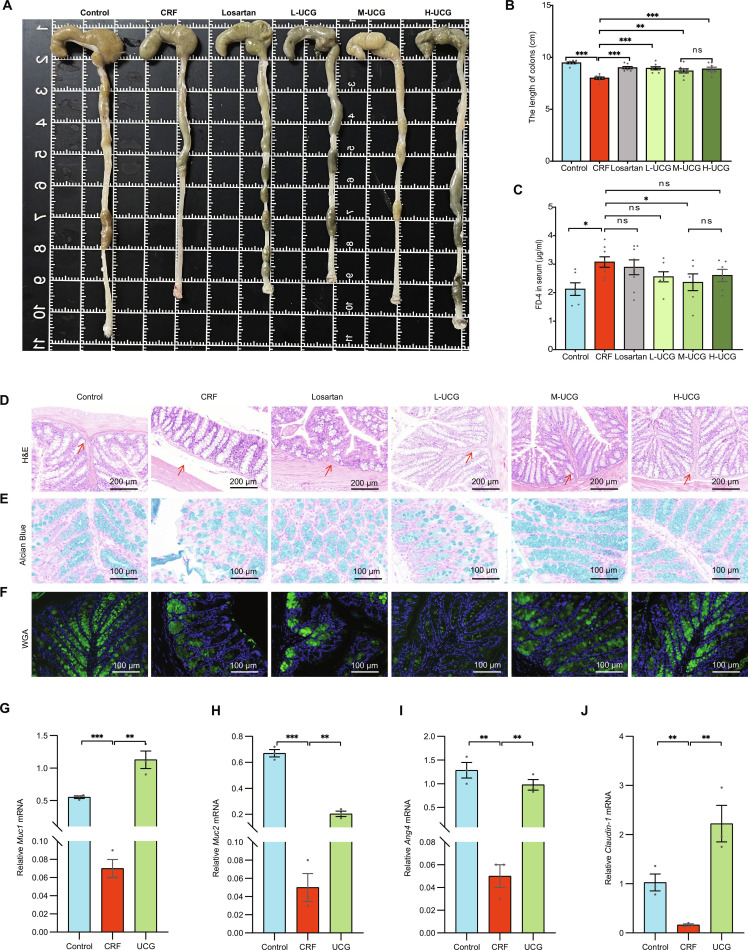
UCG restores intestinal barrier damage caused by CRF. (A) Representative images of colonic tissue. (B) Colon length. (C) Plasma levels of FD-4. (D) H&E staining of the colon, and the representative areas were highlighted by arrows (scale bar = 200 μm). (E) Alcian Blue staining of the colon (scale bar = 100 μm). (F) FITC-conjugated wheat germ agglutinin (WGA) staining of the colon (scale bar = 100 μm). (G to J) Relative mRNA levels of *Muc1*, *Muc2*, *Ang4*, and *Claudin-1* in colon tissue. *P < 0.05, **P < 0.01, ***P < 0.001.

To evaluate the impact of UCG on colonic pathological changes, we examined the colon length and tissue structure in CRF mice. Results demonstrated that compared to the normal group, CRF mice exhibited significantly shortened colon length (*P* < 0.05) (Fig. 4A and B) and damaged crypt structures, with impaired colonic tissue architecture (Fig. 4D), indicating severe colonic inflammation. Notably, treatment with medium and high doses of UCG effectively alleviated the pathological symptoms of colitis. Furthermore, to assess the effects of UCG on colonic epithelial barrier function, we evaluated goblet cell count and intestinal barrier permeability. The findings revealed that goblet cell count was significantly reduced in CRF mice (Fig. 4E and F) but was notably restored following UCG treatment. In addition, we assessed intestinal barrier permeability by measuring serum FITC-dextran levels and observed that UCG treatment significantly decreased serum FITC-dextran levels (Fig. 4C), further indicating the ability of UCG to improve intestinal barrier function. Moreover, M-UCG consistently demonstrated the most potent therapeutic effect.

Finally, we investigated the mRNA expression levels of colonic barrier-related factors. The results showed that in CRF mice, the mRNA expression levels of *Muc1* (Fig. 4G), *Muc2* (Fig. 4H), *Ang4* (Fig. 4I), and *Claudin-1* (Fig. 4J) were significantly down-regulated (*P* < 0.05). However, UCG treatment significantly up-regulated the expression of these factors (*P* < 0.05), suggesting that UCG improves colonic injury by enhancing the expression of colonic barrier-related factors. In addition, we further detected the relative mRNA expression levels of inflammatory factors in colonic tissue. In the CRF group, the mRNA expression levels of *TLR4* (Fig. S2A), *TNF-α* (Fig. S2B), *IL-1β* (Fig. S2C), and *IL-8* (Fig. S2D) were significantly up-regulated (*P* < 0.05). After UCG intervention, the expression levels were significantly down-regulated (*P* < 0.05).

In summary, the experimental results indicate that UCG alleviates colonic inflammation in CRF mice by improving colonic tissue pathology, restoring colonic epithelial barrier function, and regulating the expression of barrier-related factors and inflammatory factors. These findings further support the potential clinical value of UCG in treating chronic kidney failure accompanied by intestinal injury.

### UCG exerts its role in improving chronic kidney failure based on gut microbiota

**Fig. 5.**
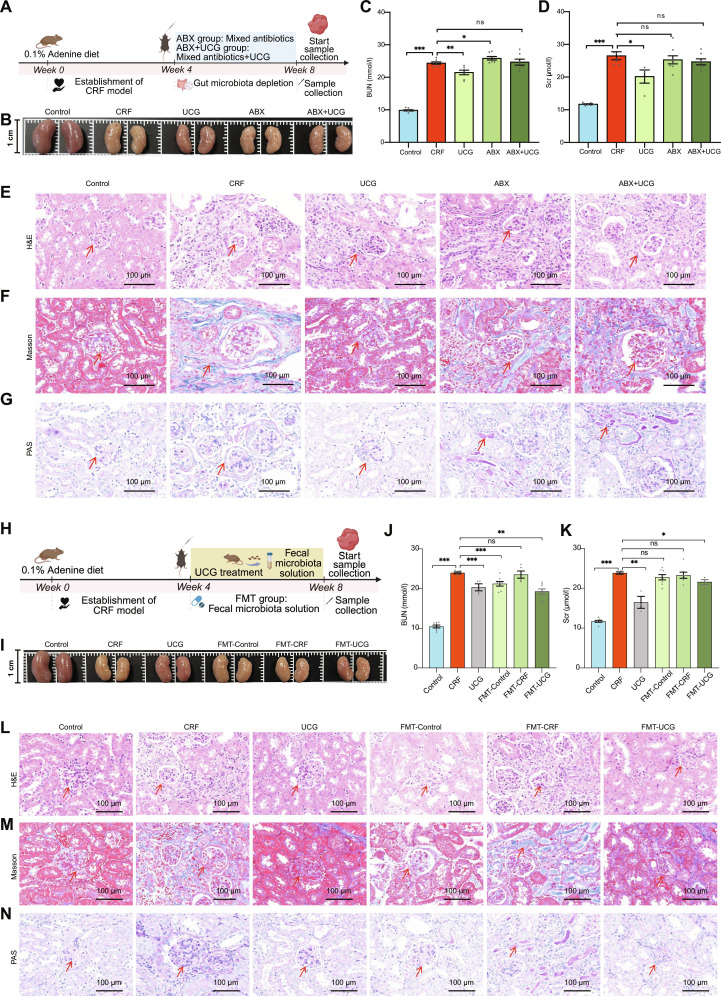
UCG intervenes with CRF through intestinal microbiota. (A) ABX experimental schematic: Mice were treated with adenine, followed by antibiotics and UCG for 4 weeks. (B) Renal morphology in the gut microbiota depletion experiment. (C) Serum BUN concentrations. (D) Scr concentrations. (E) H&E staining of the kidney, and the representative areas were highlighted by arrows (scale bar = 100 μm). (F) Masson staining of the kidney, and the representative areas were highlighted by arrows (scale bar = 100 μm). (G) PAS staining of the kidney, and the representative areas were highlighted by arrows (scale bar = 100 μm). (H) FMT experimental schematic: Mice were fed an adenine diet followed by FMT for 4 weeks. (I) Renal morphology in the fecal bacteria transplantation experiment. (J) Serum BUN concentrations. (K) Scr concentrations. (L) H&E staining of the kidney, and the representative areas were highlighted by arrows (scale bar = 100 μm). (M) Masson staining of the kidney, and the representative areas were highlighted by arrows (scale bar = 100 μm). (N) PAS staining of the kidney, and the representative areas were highlighted by arrows (scale bar = 100 μm). *P < 0.05, **P < 0.01, ***P < 0.001.

To investigate the role of gut microbiota in the improvement of CRF by UCG, we assessed the protective effect of UCG in gut microbiota-depleted mice (Fig. 5A). To confirm the depletion of gut microbiota in antibiotic-treated mice, we performed absolute quantification of microbiota in the feces of antibiotic-treated mice. The results showed that the major gut microbial phyla in the microbiota-depleted mice were significantly reduced, such as Actinomycetota, Deferribacterota, Bacteroidota, and Verrucomicrobiota (Fig. S3). The kidney appearance (Fig. 5B) and pathological features (Fig. 5E to G) in the UCG-treated group showed improvements, but the therapeutic effect was significantly lower than that in the nondepleted UCG group (*P* < 0.05). Notably, compared to the UCG group, the ABX+UCG group showed no significant improvement in CRF, as evidenced by BUN and Scr levels (Fig. 5C and D), indicating that UCG efficacy in CRF mice was substantially diminished after gut microbiota depletion. Previous results suggested that UCG’s therapeutic effects are mediated through the gut microbiota.

To further verify this, we performed FMT in CRF mice (Fig. 5H). The FMT-UCG treatment significantly alleviated kidney injury (Fig. 5I), inflammation (Fig. 5J), and fibrosis (Fig. 5K), similar to the effects observed in the UCG group (*P* < 0.05). Scr and urea nitrogen levels were also significantly reduced in the FMT-UCG group (*P* < 0.05) (Fig. 5L to N). These findings suggest that fecal microbiota from UCG-treated mice promote kidney recovery, highlighting the critical role of gut microbiota in UCG-mediated CRF improvement.

### UCG restores the *B. animalis* in CRF mice

**Fig. 6.**
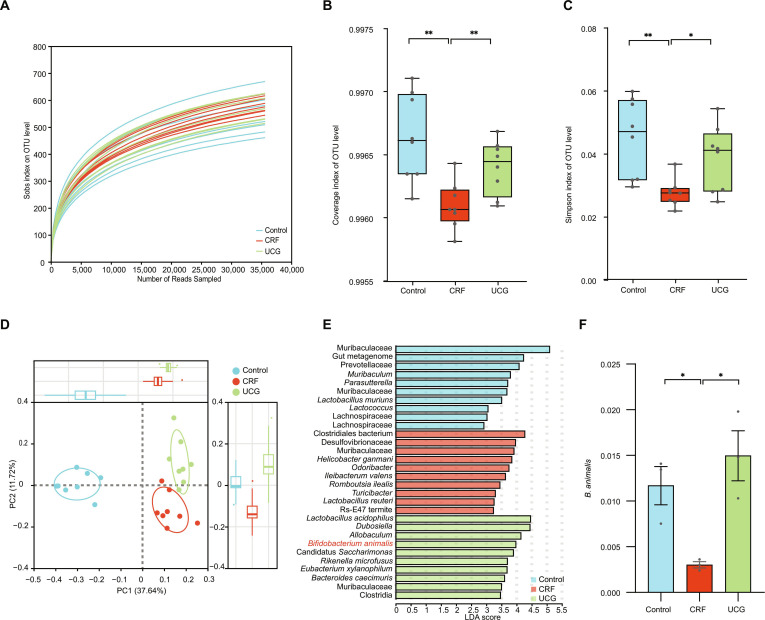
Fecal 16*S* rRNA sequencing indicated that UCG restored the intestinal flora disorder in CRF mice. (A) Grouped rarefaction curves. (B) Simpson index. (C) Coverage index. (D) PCoA based on OTUs. (E) Linear discriminant analysis (LDA). (F) Relative abundance of *B. animalis* in mice treated with UCG. *P < 0.05, **P < 0.01.

The role of gut microbiota in the improvement of CRF by UCG has been established, prompting further investigation into how UCG modulates intestinal microorganisms. To assess the impact of UCG on gut microbiota diversity and richness, we performed 16*S* rRNA sequencing on mouse intestinal contents. The results showed that the operational taxonomic unit (OTU) rarefaction curves reached a plateau (Fig. 6A), indicating good sequencing quality and reliable data. Subsequently, we used Simpson and Shannon indices to assess the diversity and richness of the gut microbiota to systematically evaluate the regulatory effects of UCG on gut microbiota diversity. The findings indicated that compared to the normal group, both Simpson and Shannon indices were significantly reduced in CRF mice (*P* < 0.05), suggesting that CRF significantly disrupted gut microbiota diversity and richness. However, following UCG treatment, the α-diversity indices significantly increased (Fig. 6B and C), indicating that UCG effectively restored gut microbiota diversity.

To further analyze the regulatory effects of UCG on gut microbiota structure, we conducted β-diversity analysis including principal component analysis (PCA) (Fig. S4) and principal coordinates analysis (PCoA) (Fig. 6D). The results demonstrated that UCG treatment significantly improved gut ecological imbalance in CRF mice (*P* < 0.05), indicating that UCG modulated the overall structure of gut microbiota and restored gut ecological balance. At the phylum level, we detected that Actinomycetota, Bacteroidota, Firmicutes, and Desulfobacterota were the predominant gut bacterial phyla across all groups of mice (Fig. S5). In addition, we performed linear discriminant analysis effect size (LEfSe) analysis to identify the dominant gut microbiota in UCG-treated mice. The findings revealed that *B. animalis* was the dominant gut microbiota in UCG-treated mice (Fig. 6E). Furthermore, *B. animalis* abundance was significantly reduced in CRF mice (*P* < 0.05), but its abundance was significantly increased after UCG treatment (*P* < 0.05) (Fig. 6F). This result suggests that *B. animalis* played a crucial role in the therapeutic effects of UCG on CRF.

In summary, this study demonstrates that UCG improves gut ecological imbalance in CRF mice by modulating gut microbiota diversity and structure, particularly by increasing the abundance of *B. animalis*. These findings provide important experimental evidence for elucidating the mechanisms by which UCG exerts its therapeutic effects in the treatment of chronic kidney failure.

### UCG up-regulates sophoridine levels in mice with CRF

**Fig. 7.**
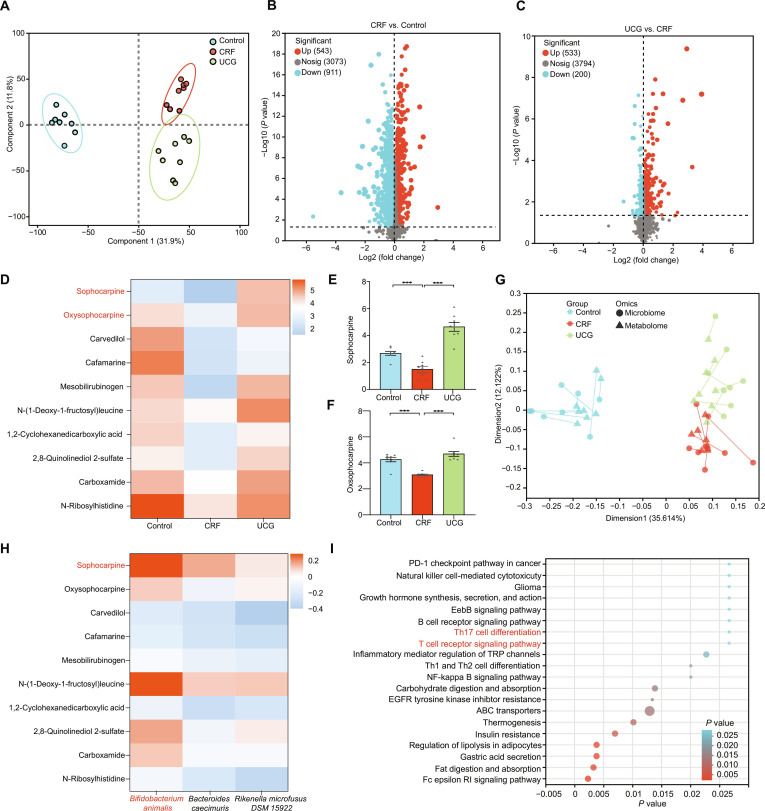
UCG regulates metabolite levels and immune cell balance to alleviate CRF. (A) Partial least squares-discriminant analysis of metabolites. (B and C) Volcano plots of differential metabolites among control, CRF, and UCG-treated mice. (D) Heatmap of differential metabolites in UCG-treated mice. (E and F) Comparison of relative intensities of sophocarpine and oxsophocarpine. (G) Procrustes analysis of microbiome versus fecal metabolome. (H) Heatmap of correlation between differential gut species and metabolites. (I) Metabolic pathways affected by UCG in CRF. ***P < 0.001.

To further investigate the gut metabolites mediating the renal protective effects of UCG in CRF, we employed metabolomics. Principal component analysis (Fig. S6) and partial least squares-discriminant analysis (Fig. 7A) revealed significant differences in the metabolic compositions among the groups. After UCG treatment, metabolites were restored to normal levels. To comprehensively understand the impact of UCG on gut metabolites, we conducted a statistical analysis of metabolite quantity across all groups. Compared to the control group, 543 metabolites were elevated, while 911 were reduced in CRF. In contrast, compared to the CRF group, 553 metabolites increased and 200 decreased after UCG treatment (Fig. 7B and C and Fig. S7).

Furthermore, to identify the primary metabolites following UCG treatment, further analysis revealed that sophoricoside and sophoricoside oxide were the predominant metabolites (Fig. 7D). Notably, these 2 metabolites showed significant elevation in the UCG-treated groups (*P* < 0.05) (Fig. 7E and F), suggesting their potential role in the renal protective effects of UCG. Research has found that sophocarpine exhibits anti-inflammatory and immune regulatory properties [27,28]. It has also been reported that sophocarpine alleviates isoproterenol-induced kidney injury by suppressing inflammation, apoptosis, oxidative stress, and fibrosis [29]. In addition, to investigate the relationship between gut microbiota and metabolites, we performed Procrustes analysis. The results indicated a significant correlation between gut microbiota and metabolites (Fig. 7G). Specifically, *B. animalis* exhibited a significant positive correlation with sophoricoside (Fig. 7H), further supporting the crucial role of gut microbiota in UCG-mediated regulation of metabolites.

Finally, to elucidate the potential molecular mechanisms underlying UCG’s regulation of metabolites, we conducted KEGG enrichment analysis. KEGG enrichment analysis indicated that UCG primarily influences immunomodulatory pathways, including Th17 cell differentiation and T cell receptor signaling (Fig. 7I). This finding suggests that UCG alleviate CRF by regulating gut microbiota and restoring immune balance.

### *B. animalis* ameliorates adenine-induced kidney injury in mice

**Fig. 8.**
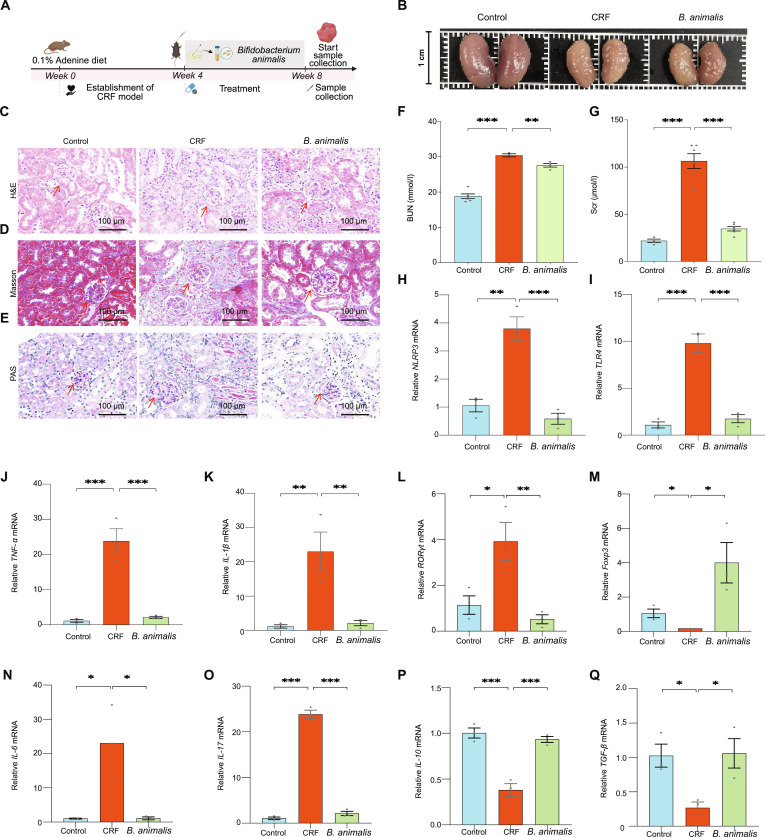
*B. animalis* modulates immunity to improve chronic kidney failure. (A) Experimental timeline: Mice were fed an adenine diet, followed by a 4-week *B. animalis* intervention. (B) Renal morphology. (C) H&E staining of the kidney, and the representative areas were highlighted by arrows (scale bar = 100 μm). (D) Masson staining of the kidney, and the representative areas were highlighted by arrows (scale bar = 100 μm). (E) PAS staining of the kidney, and the representative areas were highlighted by arrows (scale bar = 100 μm). (F) Serum BUN levels. (G) Scr levels. (H to K) mRNA expression of *NLPR3*, *TLR4*, *TNF-α*, and *IL-1β* in kidney tissue. (L) mRNA expression levels of the transcription factor *RORγt* in Th17 immune cells within kidney tissue. (M) mRNA expression levels of the transcription factor *Foxp3* in Treg immune cells within kidney tissue. (N to Q) Relative mRNA levels of *IL-6*, *IL-17*, *IL-10*, and *TGF-β* in kidney. *P < 0.05, **P < 0.01, ***P < 0.001.

To validate the role of *B. animalis* in UCG-mediated gut microbiota regulation and CRF alleviation, we conducted a *B. animalis* intervention experiment (Fig. 8A). The results demonstrated that *B. animalis* intervention significantly attenuated renal injury in CRF mice, indicating its crucial role in the renal protective effects of UCG. As expected, compared to the CRF group, the renal abnormalities in the *B. animalis* group were significantly alleviated (Fig. 8B), and pathological analysis showed that the pathological damages such as glomerular atrophy, tubular dilation, and fibrosis were notably restored (Fig. 8C to E). In addition, *B. animalis* intervention significantly reduced BUN and creatinine levels (*P* < 0.05) (Fig. 8F and G). Furthermore, to investigate the regulatory effects of *B. animalis* on inflammatory responses in CRF mice, we examined the expression levels of key inflammatory factors. Compared to the CRF group, the gene expression levels of inflammatory factors *NLPR3* (Fig. 8H), *TLR4* (Fig. 8I), *TNF-α* (Fig. 8J), and *IL-1β* (Fig. 8K) in the *B. animalis* group were significantly down-regulated (*P* < 0.05).

In terms of immune regulation, to assess the impact of *B. animalis* on immune balance in CRF mice, we measured the expression levels of transcription factors of Th17 and Treg immune cells. The mRNA expression levels of the transcription factor *RORγt* in Th17 immune cells within kidney tissue of mice in the *B. animalis* group were significantly reduced (Fig. 8L), while the mRNA expression levels of the transcription factor *Foxp3* in Treg immune cells were significantly increased (Fig. 8M), indicating that the imbalance of immune cells was significantly alleviated. In addition, further analysis of the expression changes in proinflammatory and anti-inflammatory cytokines revealed that *B. animalis* intervention mitigated renal injury and immune imbalance in CRF mice by modulating the balance of proinflammatory and anti-inflammatory cytokines. In the context of CRF, the expression levels of the proinflammatory cytokines *IL-6* (Fig. 8N) and *IL-17* (Fig. 8O) were significantly reduced following *B. animalis* treatment, while the expression levels of the anti-inflammatory cytokines *IL-10* (Fig. 8P) and *TGF-β* (Fig. 8Q) were significantly increased.

In summary, this study demonstrates that *B. animalis* ameliorates renal pathology, regulates immune balance, and suppresses inflammatory responses, thereby significantly reducing renal injury in CRF mice. These findings directly demonstrate the crucial role of *B. animalis* in UCG-mediated CRF alleviation, providing important experimental evidence for elucidating the mechanisms of action of UCG.

### UCG treatment alleviate CRF by inhibiting Th17 differentiation and enhancing Treg differentiation

**Fig. 9.**
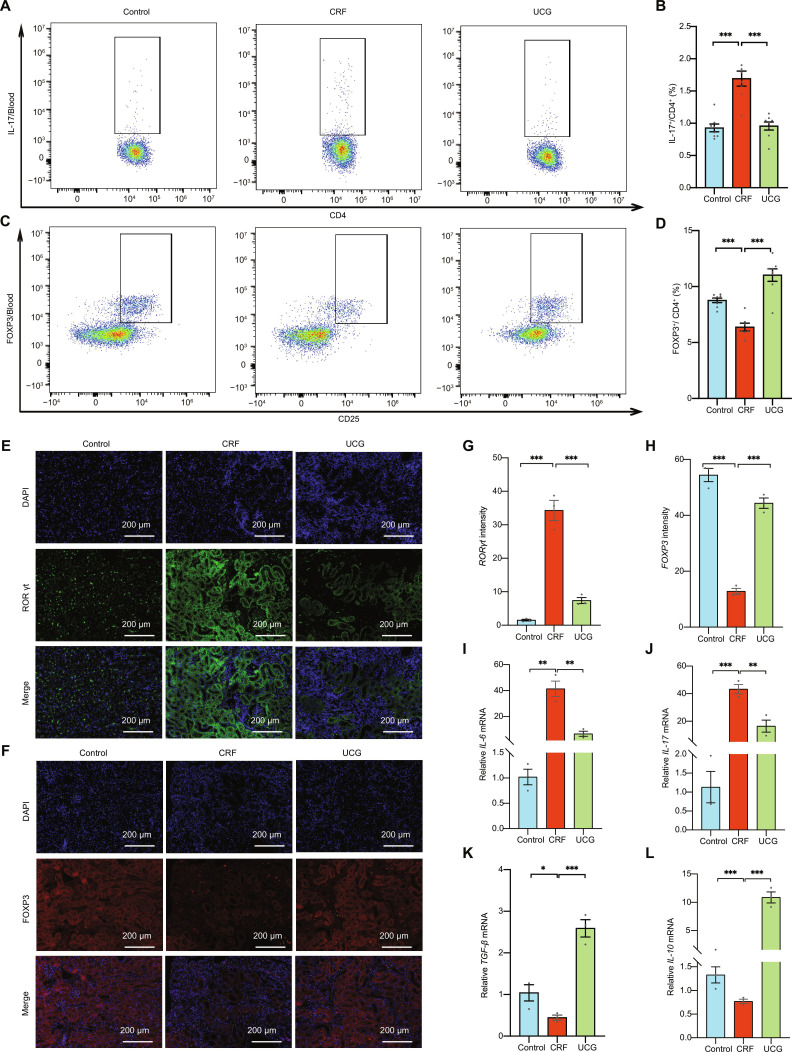
UCG regulates the balance of Th17/Treg immune cells in CRF mice. (A and B) Proportions of *IL-17*^+^ Th17 cells and their ratio among total CD4^+^ T cells in peripheral blood, assessed by flow cytometry and statistically analyzed. (C and D) Proportions of *Foxp3*^+^ Treg cells and their ratio among total CD25^+^ T cells in peripheral blood, assessed by flow cytometry and statistically analyzed. (E and G) Representative images of immunofluorescence staining of *RORγt* in kidney. (F and H) Representative images of immunofluorescence staining of *Foxp3* in kidney. (I to L) Relative mRNA levels of *IL-6*, *IL-17*, *TGF-β*, and *IL-10* in kidney. *P < 0.05, **P < 0.01, ***P < 0.001.

Based on our previous findings, we hypothesized that UCG might alleviate CRF by modulating immune cells. Therefore, to verify this hypothesis, we investigated the mechanism by which UCG improves CRF through Th17/Treg cell-mediated immune regulation. Firstly, we utilized flow cytometry to determine the counts of Th17 and Treg cells to evaluate the regulatory effects of UCG on peripheral immune cells (Fig. S8). The results showed that UCG treatment significantly reduced the number of Th17 cells (*P* < 0.05) (Fig. 9A and B) and significantly increased Treg cell counts (*P* < 0.05) (Fig. 9C and D).

Subsequently, to gain deeper insights into the specific mechanisms by which UCG influences Th17 and Treg cells, we performed immunofluorescence staining analysis of kidney tissues. The results indicated that in the CRF group, *RORγt* expression was significantly elevated (Fig. 9E and G), while *Foxp3* expression was notably reduced (Fig. 9F and H). However, UCG treatment significantly enhanced *Foxp3* expression while reducing *RORγt* levels, suggesting that UCG regulates immune responses by modulating the expression of key transcription factors involved in Th17 and Treg cell differentiation.

In addition, to assess the impact of UCG on immune-inflammatory responses in renal tissues, we measured the expression levels of relevant inflammatory factors. The results demonstrated that UCG treatment significantly suppressed the expression of proinflammatory cytokines *IL-6* and *IL-17* (*P* < 0.05) (Fig. 9I and J) while up-regulating the secretion of anti-inflammatory cytokines *TGF-β* and *IL-10* (*P* < 0.05) (Fig. 9K and L).

## Discussion

Kidney failure is widely recognized as the most severe outcome of CKD, with symptoms primarily resulting from complications due to impaired renal function [30]. In advanced cases, dialysis or renal transplantation often remain the only viable treatment options. Therefore, identifying effective pharmacological interventions to halt CRF progression and elucidating their underlying mechanisms remain a major challenge in clinical practice. UCG, a traditional Chinese herbal formulation, have been used to treat CRF for more than 2 decades [18]. However, their pharmacological basis and mechanisms of action remain poorly understood.

The gut microbiota plays a critical role in maintaining host metabolic and immune homeostasis. Exploring the gut microbiota as a therapeutic target has become a key strategy for elucidating the mechanisms of TCM [31–34]. Numerous studies have implicated gut microbial dysbiosis as a pathogenic factor in CKD and its complications. Conversely, restoration of microbial homeostasis may provide an effective intervention to mitigate CKD progression. Based on this evidence, we hypothesized that UCG exerts its renoprotective effects by modulating gut microbiota composition and associated metabolites.

Our findings demonstrated that UCG significantly alleviated renal injury in CRF mice, as reflected by improvements in biochemical indicators, histopathological observations, and inflammatory factor profiles. Further experiments using medium-dose UCG are ongoing to refine these results. UCG treatment also increased colon length, alleviated mucosal damage, and enhanced goblet cell numbers and mucin secretion while reducing intestinal permeability. Because goblet cells secrete mucins that maintain intestinal barrier integrity [35], these findings indicate that UCG effectively mitigates intestinal barrier disruption induced by CRF.

The essential role of the gut microbiota in mediating the therapeutic efficacy of UCG was further confirmed. UCG exhibited minimal benefit in microbiota-depleted mice, whereas FMT—a clinically recognized therapeutic strategy [36,37] transferred from UCG-treated donors—significantly alleviated renal injury. Previous studies have linked CRF onset to gut microbial dysbiosis, with microbiota-derived metabolites acting as key mediators of disease progression [10]. Consistent with this, 16*S* rRNA sequencing and metabolomic analyses revealed that UCG restored microbial balance, prominently increasing *B. animalis* abundance and its correlated metabolite, sophocarpine, both of which were markedly elevated in the UCG group. KEGG pathway enrichment analysis identified major metabolic processes, including the T cell receptor signaling pathway and Th17 cell differentiation. Given that gut microbiota-derived metabolites regulate intestinal barrier function and immune responses [38], these findings suggest that UCG alleviates CRF through microbiota–metabolite–immune interactions that restore immune cell balance.

Immune dysregulation contributes to renal dysfunction and accelerates CRF progression [39]. Among immune cell populations, effector Th17 cells and Treg cells—2 distinct CD4^+^ subsets—play pivotal roles in modulating renal inflammation. Treg cells (CD4^+^CD25^+^) suppress autoreactive T cells and maintain immune tolerance [40,41], while also mitigating tissue inflammation and promoting repair [42]. Thus, UCG may exert renoprotective effects by rebalancing Th17/Treg activity and preserving immune homeostasis.

*Bifidobacterium* species are known to modulate gut microbial composition and immune function. For example, *Bifidobacterium longum* CCFM1029 enhances tryptophan metabolism, promoting indole-3-carbinol production that activates aryl hydrocarbon receptor-mediated immune responses, thereby ameliorating atopic dermatitis [43]. Similarly, *Bifidobacterium longum* suppresses food allergy responses through lipid metabolism [44]. In clinical studies, patients with CKD exhibit a marked reduction in *B. animalis* abundance [16], consistent with our observation of decreased *Bifidobacterium* levels in CRF mice. Restoration of *B. animalis* in UCG-treated mice alleviated gut dysbiosis, reduced BUN levels, and mitigated kidney injury [45].

*Bifidobacterium* plays a crucial role in maintaining immune homeostasis [46]. *B. animalis* exhibits clinically relevant properties, including immune modulation, epithelial adherence, and reinforcement of intestinal barrier function [47]. Mechanistically, *B. animalis* activates macrophages and dendritic cells, promotes Treg differentiation, and enhances B cell function, collectively regulating both innate and adaptive immunity. In addition, *B. animalis* facilitates the colonization and function of commensal bacteria, improving the metabolic and immune profile of kidney disease patients [48]. Sophocarpine, the signature metabolite associated with *B. animalis*, possesses potent anti-inflammatory and antioxidant properties [48], alleviating renal injury by restoring immune balance [29]. It also exhibits immunosuppressive potential in autoimmune disease models, including rheumatoid arthritis and systemic lupus erythematosus. Li et al. [49] reported that sophora fruit extract attenuates lupus nephritis by inhibiting the NLRP3/NF-κB signaling pathway.

Flow cytometry analyses revealed that UCG treatment restored the Th17/Treg balance in CRF mice, supporting the hypothesis that this axis mediates its therapeutic effect. *RORγt* and *Foxp3* serve as transcriptional markers for Th17 and Treg cells, respectively. Th17 cells secrete *IL-17*, which activates neutrophils and induces macrophage production of *IL-1β* and *TNF-α*, thereby promoting renal inflammation [50]. Conversely, Treg cells release anti-inflammatory cytokines *IL-10* and *IL-17*, which suppress excessive immune activation and limit tissue damage. Under combined stimulation by *IL-6* and *TGF-β*, *IL-6* promotes Th17 differentiation via signal transducer and activator of transcription 3 phosphorylation. In this study, UCG treatment normalized *Foxp3* and *RORγt* expression in kidney tissues, reduced *IL-6* and *IL-17* levels, and elevated *TGF-β* and *IL-10* levels relative to CRF controls. Collectively, these findings demonstrate that UCG ameliorates CRF by restoring Th17/Treg balance through gut microbiota modulation.

## Conclusion

In summary, UCG alleviates CRF by enhancing *B. animalis* abundance and sophocarpine levels, which together reestablish Th17/Treg immune balance. This study elucidates the role of gut microbiota in regulating renal immune dysregulation, providing new insights into the pathophysiology of chronic kidney failure. Our findings offer experimental and theoretical evidence supporting microbiota-targeted therapies for CRF and related disorders. Moreover, the identification of the material basis and mechanism of UCG action provides a foundation for the development of novel therapeutics and optimized treatment strategies. Overall, this study advances the mechanistic understanding of UCG, reinforces its clinical potential, and contributes to the modernization of TCM for chronic kidney failure management.

## Data Availability

The data are available from the corresponding author on reasonable request.
