# Supplementary material for: Uremic Clearance Granules Regulate Immune Equilibrium via Gut Microbiome to Alleviate Chronic Renal Failure
Source: Biomater Res. 2026 Mar 30;30:0342. doi: 10.34133/bmr.0342 (PMC13033834; doi:10.34133/bmr.0342)
Supplement: Supplementary 1 — Figs. S1 to S8 Tables S1 to S3 [file bmr.0342.f1.docx]

**Supporting information**

**Uremic Clearance Granules Regulate Immune Equilibrium *via* Gut Microbiome to** **Alleviate Chronic Renal Failure**

Qian Huang^1,^ ^#^, Zhuowen Liang^1, #^, Yuqing Cui^1, #^, Jianxin Diao^3^, Tianshu Zhou^5^, Lei Shi^1^, Zhixin Deng^1^, Rushang Wang^4^, Haitao Yuan^1,2^, Kun Chen^1, 2^, Ying Du^4, *^, Ali Chen^2, *^, Jiayun Chen^1, 2, *^ and Wei Xiao^1, *^

^1^Key Laboratory of Glucolipid Metabolic Disorder, Ministry of Education, Guangdong Pharmaceutical University, Guangzhou, Guangdong, 510006, China.

^2^Center for Drug Research and Development, Guangdong Provincial Key Laboratory of Pharmaceutical Preparations Research and Evaluation, Guangdong Pharmaceutical University, Guangzhou, 510006, China.

^3^School of traditional Chinese medicine, Southern Medical University, Guangzhou, Guangdong, China, 510515.

^4^Consun Pharmaceutical Group Limited, Guangzhou, Guangdong, 510006, China.

^5^Department of Faculty of Education, City University of Hong Kong, Hong Kong, China

^#^ These authors contributed equally to this work.

*Corresponding authors: Wei Xiao (xw7688@smu.edu.cn); Jiayun Chen ([chenjiayun@gdpu.edu.cn](mailto:chenjiayun@gdpu.edu.cn)); Ali Chen ([chenali@gdpu.edu.cn](mailto:chenali@gdpu.edu.cn)); Ying Du (dr.du@chinaconsun.com)

**Materials and Methods**

1. **The experimental methods of fecal non-targeted metabolome** **experimental methods**

Untargeted m etabolomic analyses were performed on the fecal samples via the liquid chromatography-tandem mass spectrometry (UHPLC-MS/MS). 50 mg of fecal sample was weighted to an EP tube, and 400 μL extract solution (methanol: water = 4: 1, with L-2-chlorophenylalanine internal standard mixture) was added. Then the samples were homogenized at 50 Hz for 6 min and sonicated for 30 min in an ice water bath. Then the samples were incubated for 30 min at -20°C and centrifuged at 13000 rpm for 15 min at 4°C. The resulting supernatant was transferred to a fresh glass vial for analysis. The quality control (QC) sample was prepared by mixing an equal aliquot of the supernatants from all of the samples. The UHPLC system (Vanquish, Thermo Fisher Scientific) with UPLC HSST3 column (100 mm × 2.1 mm i.d., 1.8 µm) were coupled to Q Exactive HF-X mass spectrometer (Orbitrap MS, Thermo). Helium was used as a carrier gas at a constant flow rate through the column. The samples were analyzed in a random sequence. The ESI source conditions were set as following: sheath gas flow rate as 50 psi, Aux gas flow rate as 13 psi, capillary temperature 425°C, collision energy as 20/40/60 in NCE mode, spray Voltage as 3.5kV (positive) or -3.5kV (negative), full MS resolution as 60000, MS/MS resolution as 7500, respectively.

The acquired data were imported to Progenesis QI (Waters, Corporation，Milford, USA) where multivariate statistical analysis including partial least-squares discriminant analysis (PLS-DA) were performed. The differential metabolites were determined by the combination of the variable importance in the projection (VIP) value (> 1) of the PLS-DA model and the P values (<0.05) from two-tailed Student’s t test on the normalized peak intensities. Fold change was calculated as a binary logarithm of the average normalized peak area ratio between the two groups. The structural identification of differential metabolites was performed using HMDB (http://www.hmdb.ca/) and Metlin. Pathway analysis was conducted using MetaboAnalyst 5.0. Python packages “SciPy. Stats” was used to perform enrichment analysis to obtain the most relevant biological pathways.

1. **Gut microbiota profiling by** **16S rRNA sequencing**

Bacterial DNA from fecal samples was extracted using the Fast Pure Stool DNA Isolation Kit (MJYH, shanghai, China) and quantified for concentration adjustment. PCR was performed on the aliquoted DNA using the V4 region of the 16S rRNA gene (515 F and 806R). A standard thermocycler protocol was used: 95 °C for 3 min, followed by 27 cycles of 30 s at 95 °C, 30 s at 55 °C, and 30 s at 72 °C, with a final 10 min at 72 °C and hold at 4 °C. Amplifications were purified using the QIAquick PCR purification kit (Qiagen). Purified 16S rRNA amplicons were pooled in equimolar amounts, and the amplicon size was determined by an NextSeq 2000 PE300 (Illumina, Inc). Pools were sequenced with a 600-cycle kit on the Illumina MiSeq (Illumina Inc.). Raw fast files were demultiplexed and quality-filtered using QIIME (Quantitative Insights into Microbial Ecology, version 4.2; as previously described, and the sequences were picked against a high-quality 16S rRNA sequence from the Green Genes database after trimming the primer, barcode, and chimeras. Operational taxonomic units (OTUs) were picked at 97% similarity cutoff using UPARSE, and chimeric sequences were identified and removed using UCHIME. Alpha diversity was estimated on the basis of the gene profile of each sample according to the Coverage and Simpson index, while Beta-diversity was estimated by calculating Bray–Curtis dissimilarity between samples. Total sum scaling was used for data normalization and two variables were used in PCoA plotting of bacterial OTU in fecal samples.

**Supporting figures**

**
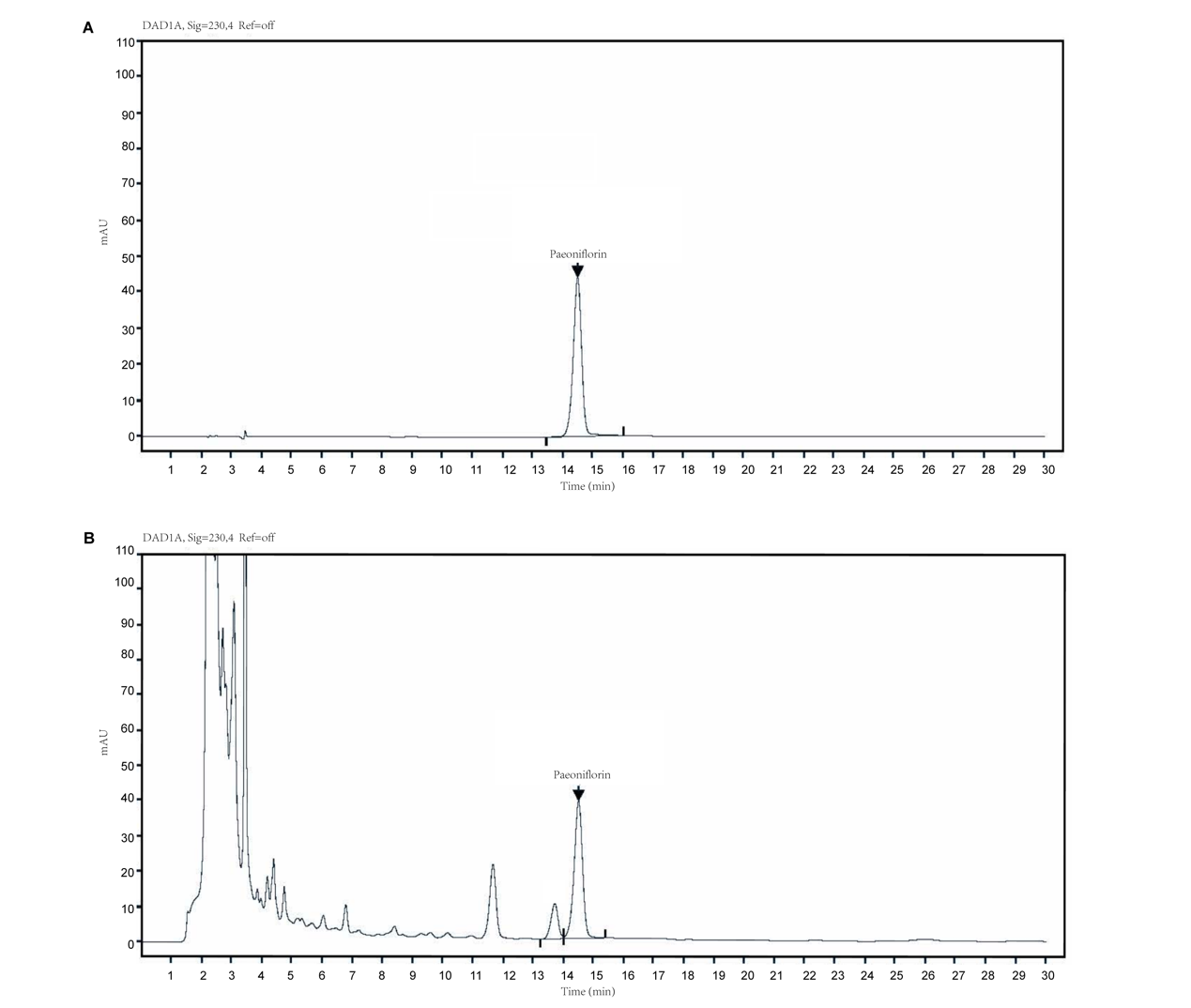
**

**Fig. S1.** LC-MS/MS analysis of UCG granules. (A) Paeoniflorin standard solution. (B) UCG test solution.


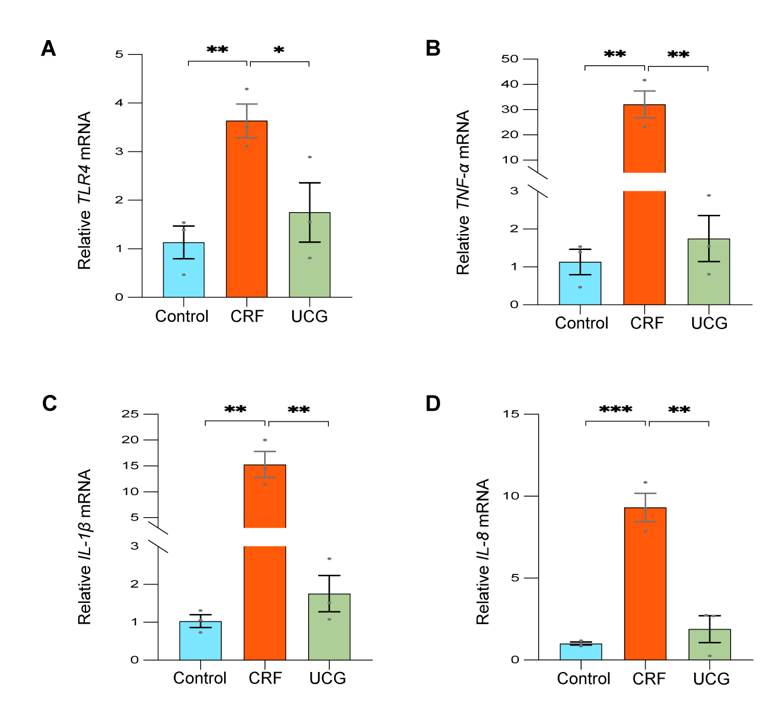


**Fig. S2.** mRNA expression of *TLR4* (A), *TNF-α* (B), *IL-1β* (C) *and IL-8* (D) in colon tissue. * *p* < 0.05, ** *p* < 0.01, *** *p* < 0.001, ns, not significant.


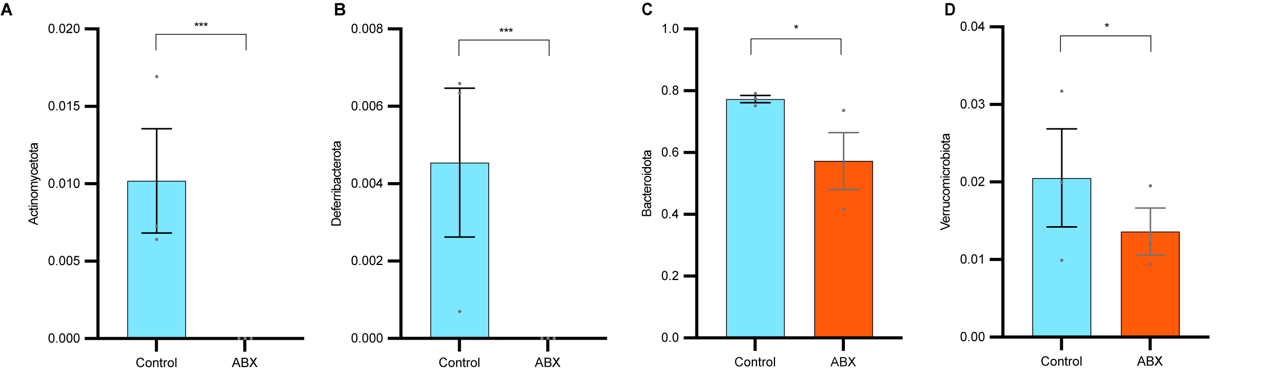


**Fig. S3.** Absolute quantification of microbiota in the feces of microbiota-depleted mice. (A) Actinomycetota. (B)Deferribacterota. (C) Bacteroidota. (D)Verrucomicrobiota. * *p* < 0.05, ** *p* < 0.01, *** *p* < 0.001, ns, not significant.


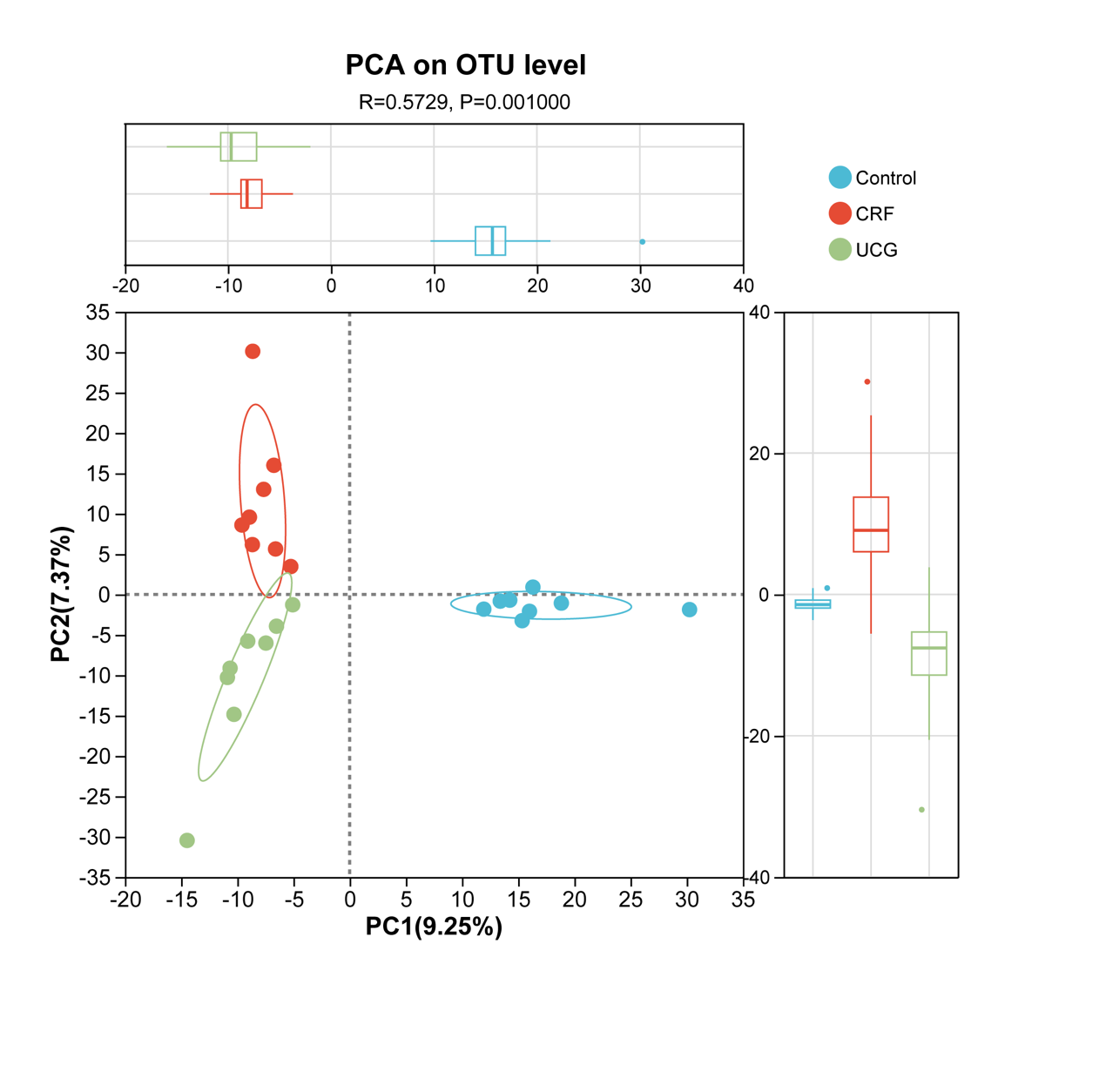


**Fig. S4.** PCA analysis based on OTUs in fecal 16S rRNA sequencing.


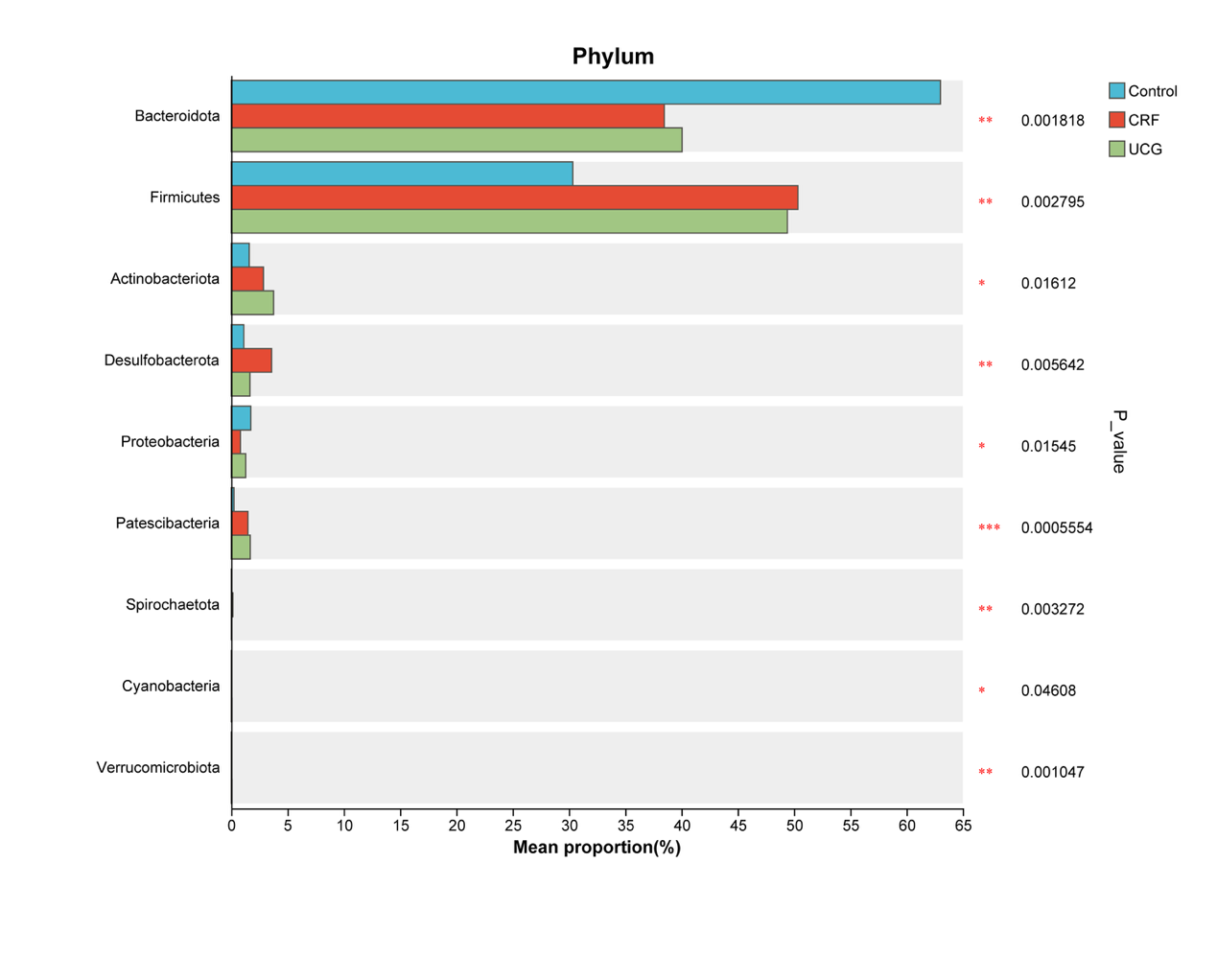


**Fig. S5.** Multi-species comparison at the phylum level in fecal 16S rRNA sequencing.


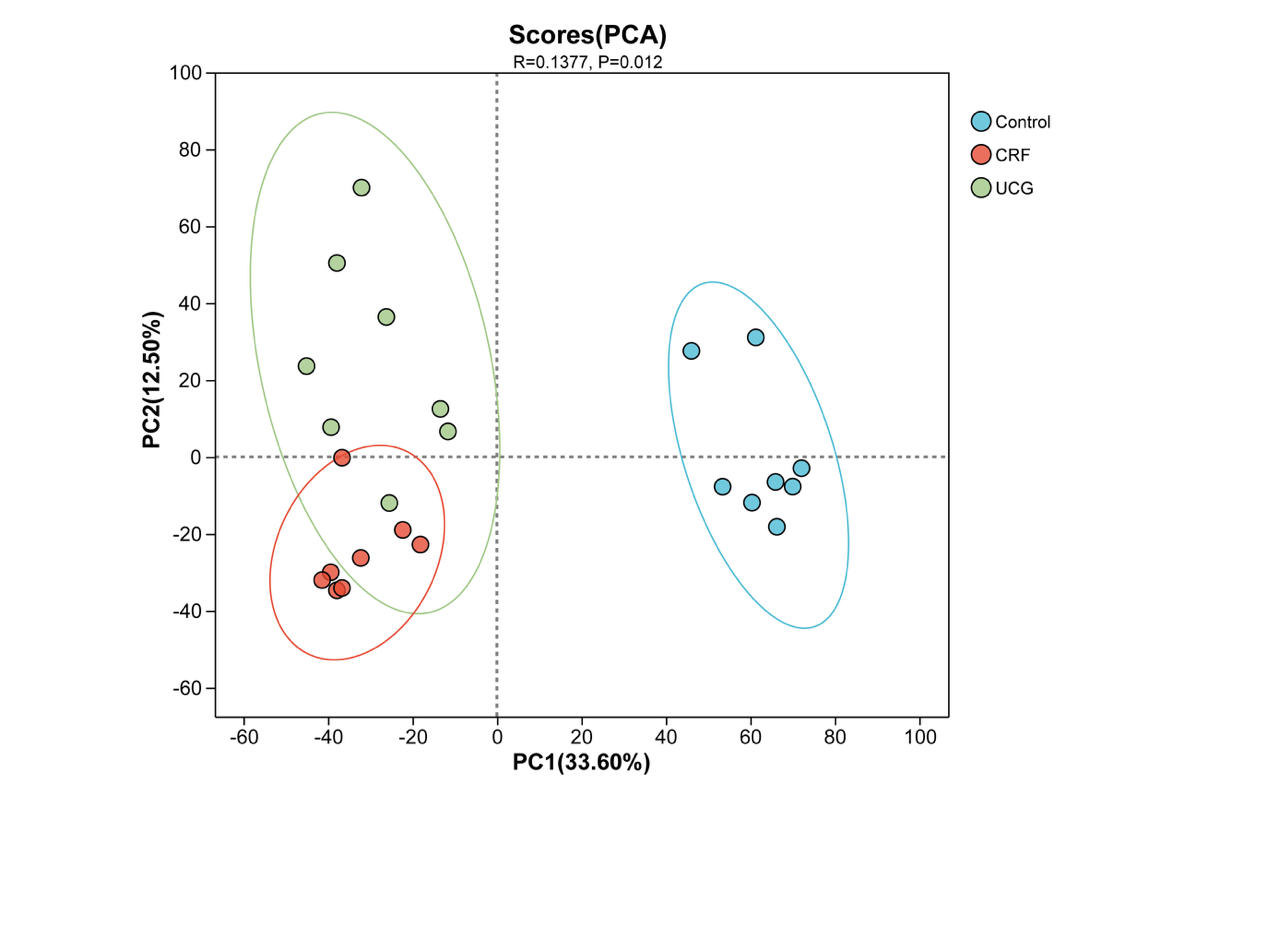


**Fig. S6.** PCA score plots of fecal metabolites.


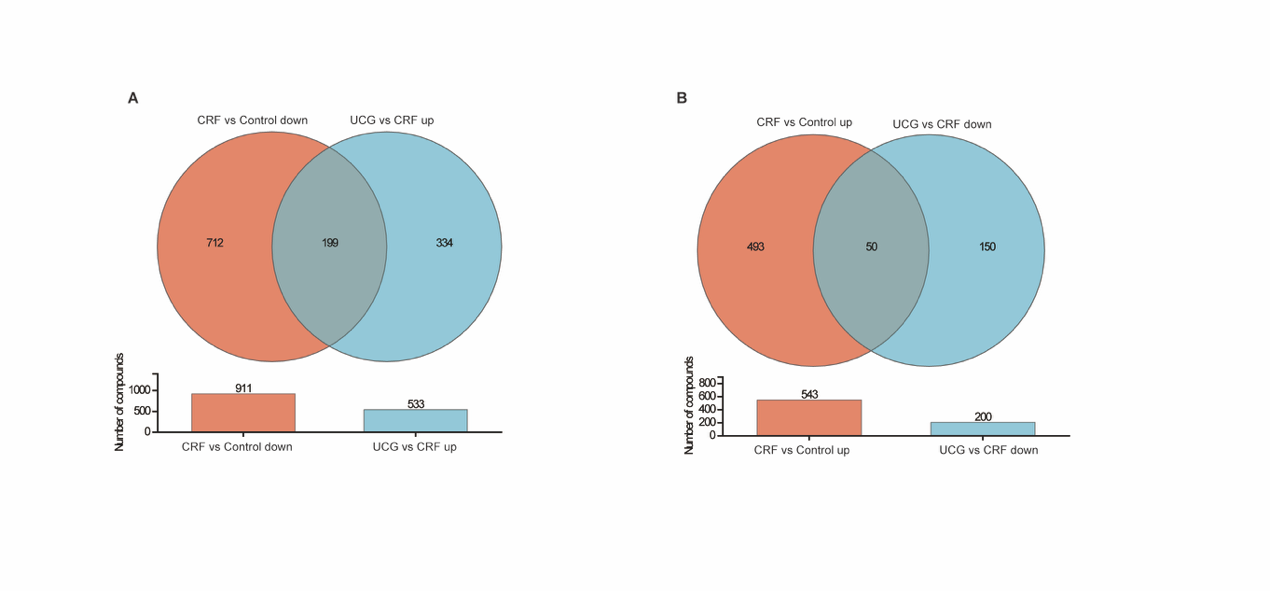


**Fig. S7.** Venn plots of differential metabolites among control, CRF, and UCG-treated mice.


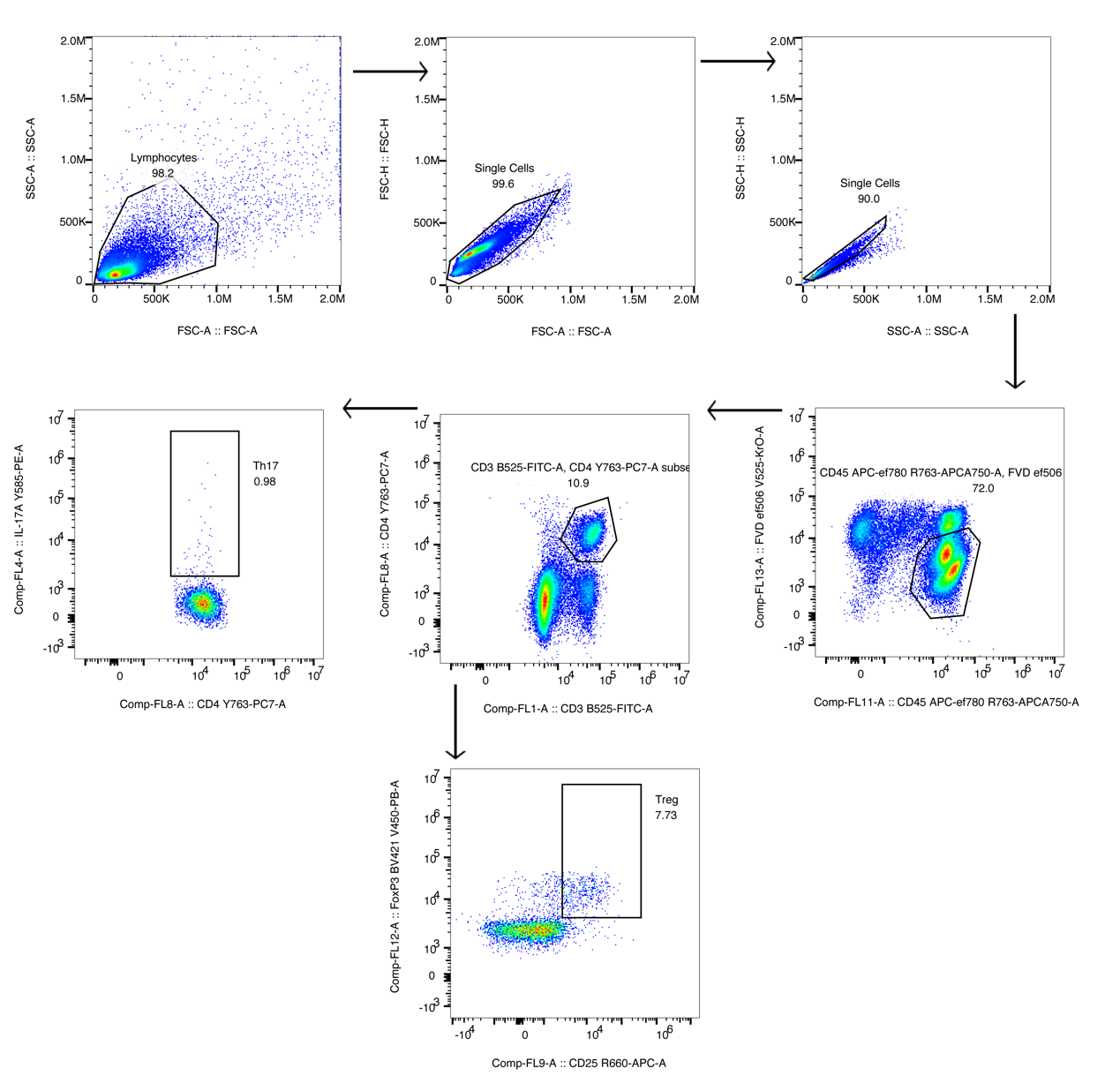


**Fig. S8.** Analytical procedure for Th17 and Treg cell identification by flow cytometry.

**Supplementary tables**

Table S1. The composition of traditional Chinese medicine ingredients

in UCG Compound Granules

| Common name | Latin name | Plant family |
| --- | --- | --- |
| Rhubarb | Rheum palmatum | Polygonaceae |
| Astragalus | Astragalus membranaceus | Fabaceae |
| Licorice | Glycyrrhiza uralensis | Fabaceae |
| Mulberry Bark | Morus aiba | Moraceae |
| Sophora | Sophora flavescens | Fabaceae |
| Ginseng | Panax ginseng | Araliaceae |
| White Peony | Peaonia lactiflora | Paeoniaceae |
| Poria | Wolfiporia extensa | Polygonaceae |
| Polygonum. Multiflorum | Polygonum multiflorum | Polygonaceae |
| White Peony Root | Paeonia lactiflora | Paeoniaceae |
| White Chrysanthemum | Chrysanthemum morifolium | Compositae |
| Szechuan Lovage | Ligusticum chuanxiong | Umbelliferae |
| Salvia | Salvia miltiorrhiza | Lamiaceae |
| Pinellia | Pinellia ternata | Araceae |
| Plantain | Plantago asiatica | Plantaginaceae |
| Bupleurum | Bupleurum chinense | Umbelliferea |

Table S2. Primer sequences

| Genes | Forward primer (5′-3′) | Reverse primer (3′-5′) |
| --- | --- | --- |
| NLRP3 | CCATCAATGCTGCTTCGACA | GAGCTCAGAACCAATGCGAG |
| TLR4 | AGGCAGCAGGTGGAATTGTA | GGTCCAAGTTGCCGTTTCTT |
| TNF-α | GCCTATGTCTCAGCCTCTTCT | TTGTGAGTGTGAGGGTCTGG |
| IL-1β | GTCTTTCCCGTGGACCTTC | ATCTCGGAGCCTGTAGTGC |
| Muc1 | TACCAAAGTGACGTCAGGCT | ATTGACTTGGCACTGAAGGC |
| Muc2 | TGACAATGTGCCCAGAGAGT | AGCTTTGCATCGTTTGGTGT |
| Ang4 | GGAAAGAAAGCTAACCTCGCC | GCGTACAAGTGGTGATCTGG |
| Claudin-1 | CCCCATCAATGCCAGGTATG | GGTGTTGGCTTGGGATAAGG |
| IL-6 | CACAGAGGATACCACTCCCAACAGA | ACAATCAGAATTGCCATTGCACAAC |
| IL-17 | AACACTGAGGCCAAGGAC | CGTGGAACGGTTGAGGTAG |
| TGF-β1 | TTGCTTCAGCTCCACAGAGA | TGGTTGTAGAGGGCAAGGAC |
| IL-10 | GCTGGACAACATACTGCTAACCG | CACAGGGGAGAAATCGATGACAG |

Table S3. Blood-borne constituents’ characterization of UCG.

| Metabolites | Chemical formula | m/z |
| --- | --- | --- |
| Guanine | C_5_H_5_N_5_O | 152.0571 |
| Dalbergin | C_16_H_12_O_4_ | 267.0659 |
| Emodin | C_15_H_10_O_5_ | 269.0452 |
| Oxysophoridine | C_15_H_24_N_2_O_2_ | 265.1914 |
| Formononetin | C_16_H_12_O_4_ | 269.0813 |
| Flazine | C_17_H_12_N_2_O_4_ | 309.0873 |
| 8-Prenyldaidzein | C_20_H_18_O_4_ | 323.1258 |
| Gardoside | C_16_H_22_O_10_ | 373.1137 |
| Luteolin 7-O-glucuronide | C_21_H_18_O_12_ | 461.0722 |
| 18alpha-Glycyrrhetinic acid | C_30_H_46_O_4_ | 469.3323 |
| Wogonin 7-glucuronide | C_22_H_20_O_11_ | 461.1082 |
| Glycyrrhetinic acid | C_30_H_46_O_4_ | 471.3478 |
| Albiflorin | C_23_H_28_O_11_ | 481.1714 |
| Luteolin 7,3'-diglucuronide | C_27_H_26_O_18_ | 639.1204 |
| 6-Hydroxyapigenin 7-glucuronosyl-(1->2)-glucuronide | C_27_H_26_O_18_ | 639.1203 |
